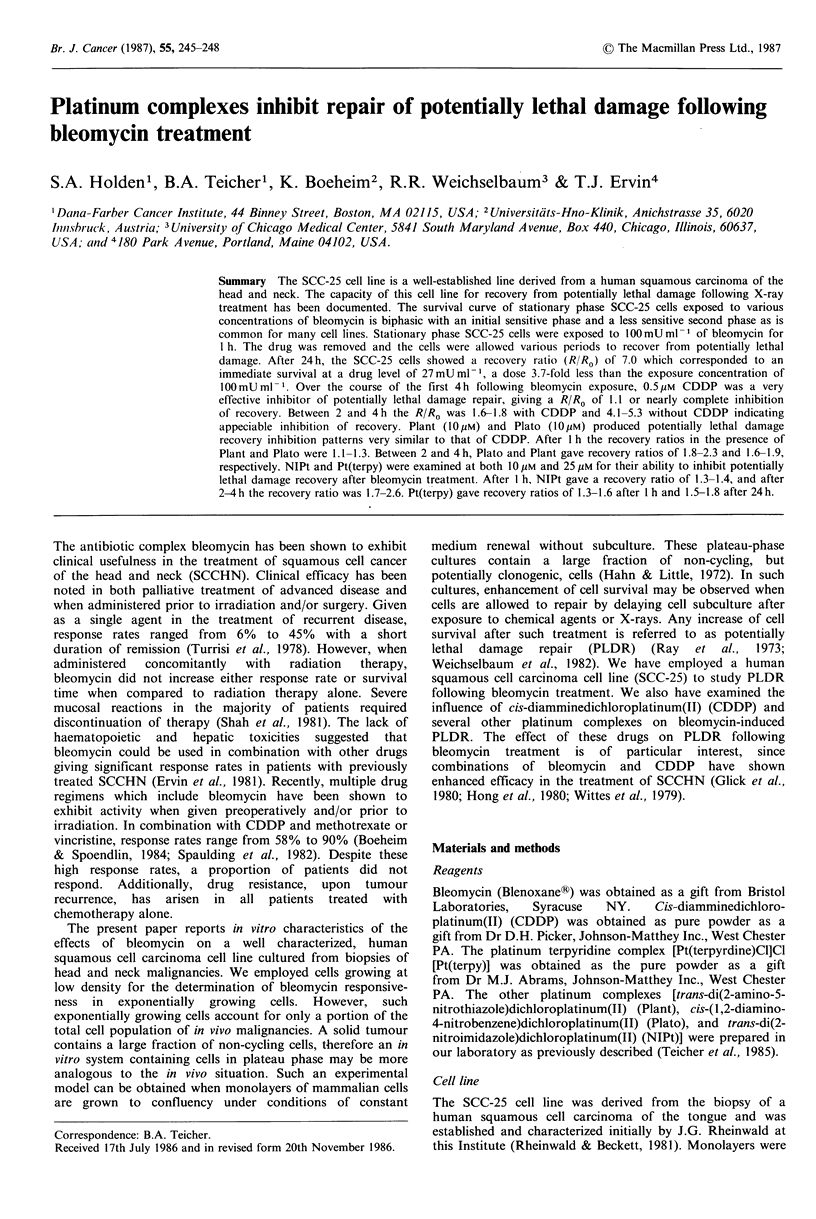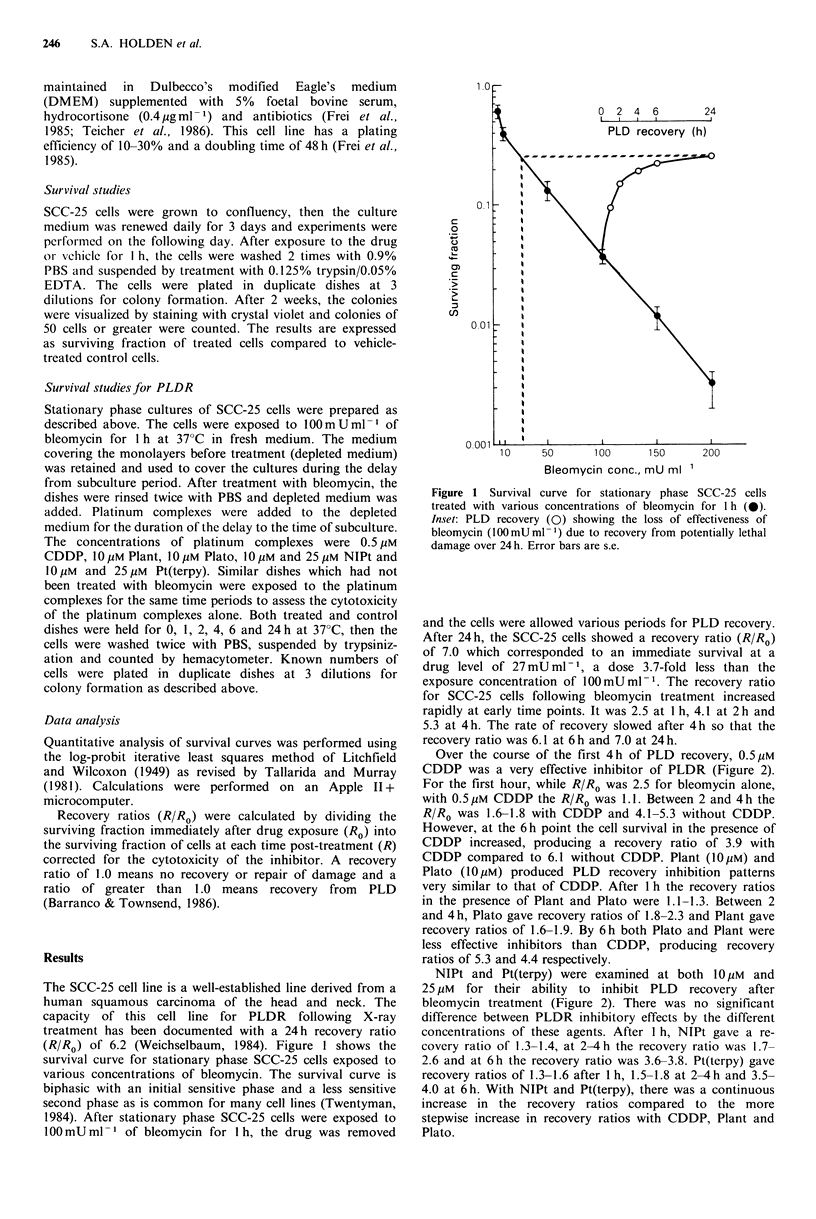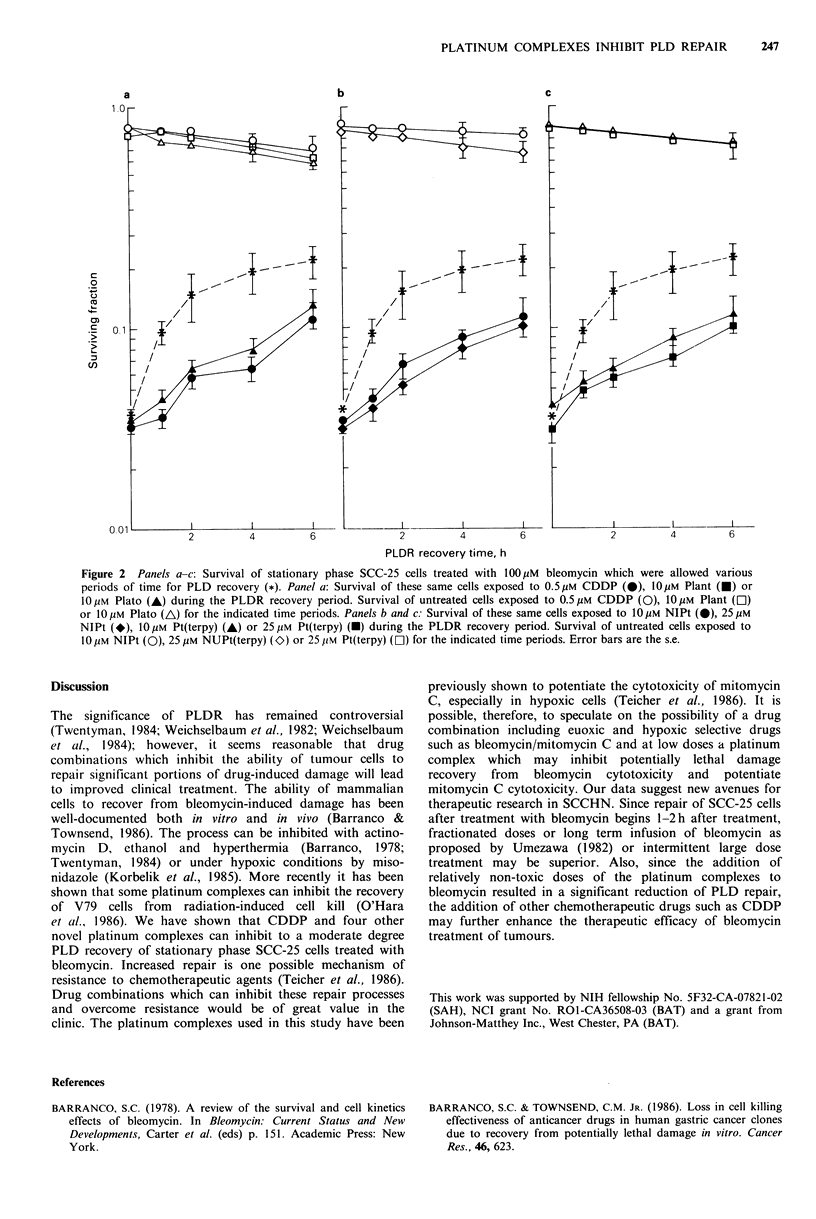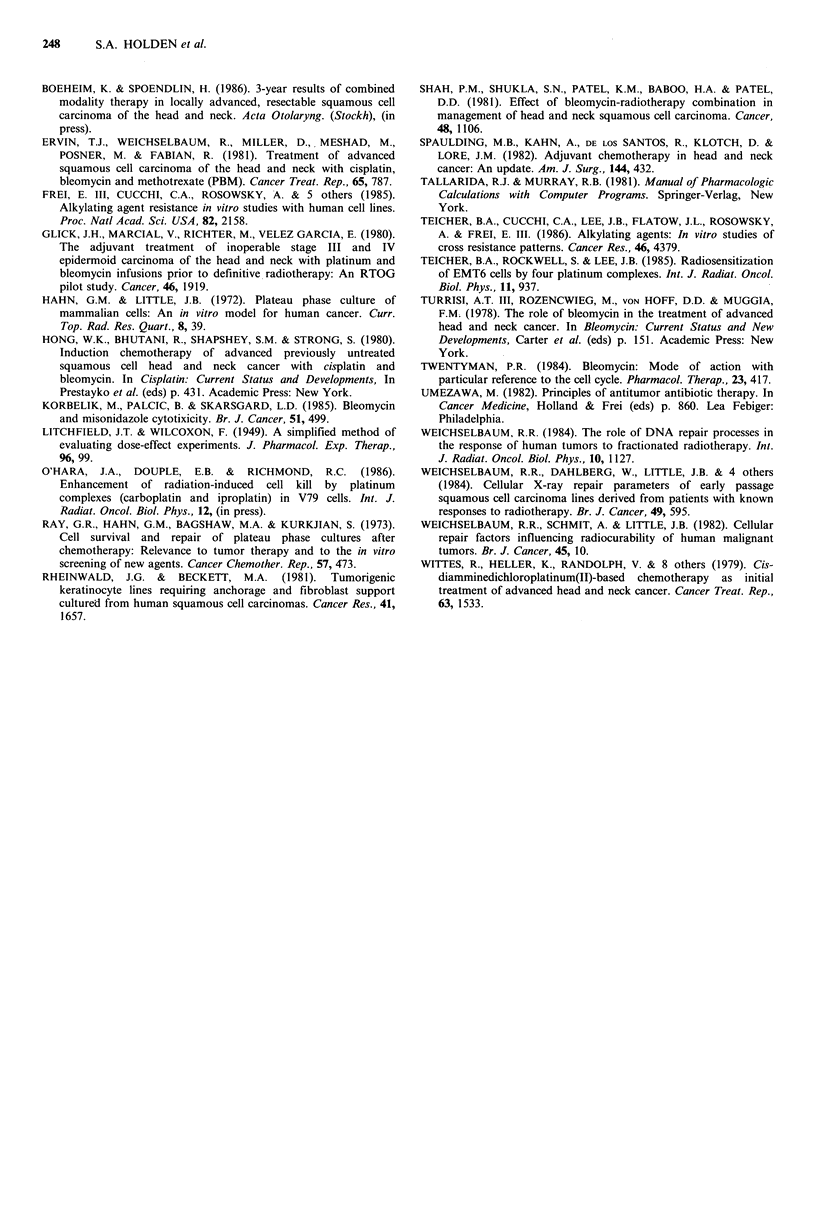# Platinum complexes inhibit repair of potentially lethal damage following bleomycin treatment.

**DOI:** 10.1038/bjc.1987.47

**Published:** 1987-03

**Authors:** S. A. Holden, B. A. Teicher, K. Boeheim, R. R. Weichselbaum, T. J. Ervin

## Abstract

The SCC-25 cell line is a well-established line derived from a human squamous carcinoma of the head and neck. The capacity of this cell line for recovery from potentially lethal damage following X-ray treatment has been documented. The survival curve of stationary phase SCC-25 cells exposed to various concentrations of bleomycin is biphasic with an initial sensitive phase and a less sensitive second phase as is common for many cell lines. Stationary phase SCC-25 cells were exposed to 100 mU ml-1 of bleomycin for 1 h. The drug was removed and the cells were allowed various periods to recover from potentially lethal damage. After 24 h, the SCC-25 cells showed a recovery ratio (R/R0) of 7.0 which corresponded to an immediate survival at a drug level of 27 mU ml-1, a dose 3.7-fold less than the exposure concentration of 100 mU ml-1. Over the course of the first 4 h following bleomycin exposure, 0.5 microM CDDP was a very effective inhibitor of potentially lethal damage repair, giving a R/R0 of 1.1 or nearly complete inhibition of recovery. Between 2 and 4 h the R/R0 was 1.6-1.8 with CDDP and 4.1-5.3 without CDDP indicating appreciable inhibition of recovery. Plant (10 microM) and Plato (10 microM) produced potentially lethal damage recovery inhibition patterns very similar to that of CDDP. After 1 h the recovery ratios in the presence of Plant and Plato were 1.1-1.3. Between 2 and 4 h, Plato and Plant gave recovery ratios of 1.8-2.3 and 1.6-1.9, respectively. NIPt and Pt(terpy) were examined at both 10 microM and 25 microM for their ability to inhibit potentially lethal damage recovery after bleomycin treatment. After 1 h, NIPt gave a recovery ratio of 1.3-1.4, and after 2-4 h the recovery ratio was 1.7-2.6. Pt(terpy) gave recovery ratios of 1.3-1.6 after 1 h and 1.5-1.8 after 24 h.


*Br. J. Cancer* (1987), **55**, 245–248 © The Macmillan Press Ltd., 1987

### Platinum complexes inhibit repair of potentially lethal damage following bleomycin treatment

S.A. Holden[1], B.A. Teicher[1], K. Boeheim[2], R.R. Weichselbaum[3] & T.J. Ervin[4]

[1]*Dana-Farber Cancer Institute, 44 Binney Street, Boston, MA 02115, USA;* [2]*Universitäts-Hno-Klinik, Anichstrasse 35, 6020 Innsbruck, Austria;* [3]*University of Chicago Medical Center, 5841 South Maryland Avenue, Box 440, Chicago, Illinois, 60637, USA; and* [4]*180 Park Avenue, Portland, Maine 04102, USA.*

**Summary** The SCC-25 cell line is a well-established line derived from a human squamous carcinoma of the head and neck. The capacity of this cell line for recovery from potentially lethal damage following X-ray treatment has been documented. The survival curve of stationary phase SCC-25 cells exposed to various concentrations of bleomycin is biphasic with an initial sensitive phase and a less sensitive second phase as is common for many cell lines. Stationary phase SCC-25 cells were exposed to $100\,mU\,ml^{-1}$ of bleomycin for 1 h. The drug was removed and the cells were allowed various periods to recover from potentially lethal damage. After 24 h, the SCC-25 cells showed a recovery ratio ($R/R_0$) of 7.0 which corresponded to an immediate survival at a drug level of $27\,mU\,ml^{-1}$, a dose 3.7-fold less than the exposure concentration of $100\,mU\,ml^{-1}$. Over the course of the first 4 h following bleomycin exposure, $0.5\,\mu M$ CDDP was a very effective inhibitor of potentially lethal damage repair, giving a $R/R_0$ of 1.1 or nearly complete inhibition of recovery. Between 2 and 4 h the $R/R_0$ was 1.6–1.8 with CDDP and 4.1–5.3 without CDDP indicating appeciable inhibition of recovery. Plant ($10\,\mu M$) and Plato ($10\,\mu M$) produced potentially lethal damage recovery inhibition patterns very similar to that of CDDP. After 1 h the recovery ratios in the presence of Plant and Plato were 1.1–1.3. Between 2 and 4 h, Plato and Plant gave recovery ratios of 1.8–2.3 and 1.6–1.9, respectively. NIPt and Pt(terpy) were examined at both $10\,\mu M$ and $25\,\mu M$ for their ability to inhibit potentially lethal damage recovery after bleomycin treatment. After 1 h, NIPt gave a recovery ratio of 1.3–1.4, and after 2–4 h the recovery ratio was 1.7–2.6. Pt(terpy) gave recovery ratios of 1.3–1.6 after 1 h and 1.5–1.8 after 24 h.

The antibiotic complex bleomycin has been shown to exhibit clinical usefulness in the treatment of squamous cell cancer of the head and neck (SCCHN). Clinical efficacy has been noted in both palliative treatment of advanced disease and when administered prior to irradiation and/or surgery. Given as a single agent in the treatment of recurrent disease, response rates ranged from 6% to 45% with a short duration of remission (Turrisi *et al.*, 1978). However, when administered concomitantly with radiation therapy, bleomycin did not increase either response rate or survival time when compared to radiation therapy alone. Severe mucosal reactions in the majority of patients required discontinuation of therapy (Shah *et al.*, 1981). The lack of haematopoietic and hepatic toxicities suggested that bleomycin could be used in combination with other drugs giving significant response rates in patients with previously treated SCCHN (Ervin *et al.*, 1981). Recently, multiple drug regimens which include bleomycin have been shown to exhibit activity when given preoperatively and/or prior to irradiation. In combination with CDDP and methotrexate or vincristine, response rates range from 58% to 90% (Boeheim & Spoendlin, 1984; Spaulding *et al.*, 1982). Despite these high response rates, a proportion of patients did not respond. Additionally, drug resistance, upon tumour recurrence, has arisen in all patients treated with chemotherapy alone.

The present paper reports *in vitro* characteristics of the effects of bleomycin on a well characterized, human squamous cell carcinoma cell line cultured from biopsies of head and neck malignancies. We employed cells growing at low density for the determination of bleomycin responsiveness in exponentially growing cells. However, such exponentially growing cells account for only a portion of the total cell population of *in vivo* malignancies. A solid tumour contains a large fraction of non-cycling cells, therefore an *in vitro* system containing cells in plateau phase may be more analogous to the *in vivo* situation. Such an experimental model can be obtained when monolayers of mammalian cells are grown to confluency under conditions of constant medium renewal without subculture. These plateau-phase cultures contain a large fraction of non-cycling, but potentially clonogenic, cells (Hahn & Little, 1972). In such cultures, enhancement of cell survival may be observed when cells are allowed to repair by delaying cell subculture after exposure to chemical agents or X-rays. Any increase of cell survival after such treatment is referred to as potentially lethal damage repair (PLDR) (Ray *et al.*, 1973; Weichselbaum *et al.*, 1982). We have employed a human squamous cell carcinoma cell line (SCC-25) to study PLDR following bleomycin treatment. We also have examined the influence of *cis*-diamminedichloroplatinum(II) (CDDP) and several other platinum complexes on bleomycin-induced PLDR. The effect of these drugs on PLDR following bleomycin treatment is of particular interest, since combinations of bleomycin and CDDP have shown enhanced efficacy in the treatment of SCCHN (Glick *et al.*, 1980; Hong *et al.*, 1980; Wittes *et al.*, 1979).

### Materials and methods

### Reagents

Bleomycin (Blenoxane®) was obtained as a gift from Bristol Laboratories, Syracuse NY. *Cis*-diamminedichloroplatinum(II) (CDDP) was obtained as pure powder as a gift from Dr D.H. Picker, Johnson-Matthey Inc., West Chester PA. The platinum terpyridine complex [Pt(terpyrdine)Cl]Cl [Pt(terpy)] was obtained as the pure powder as a gift from Dr M.J. Abrams, Johnson-Matthey Inc., West Chester PA. The other platinum complexes [*trans*-di(2-amino-5-nitrothiazole)dichloroplatinum(II) (Plant), *cis*-(1,2-diamino-4-nitrobenzene)dichloroplatinum(II) (Plato), and *trans*-di(2-nitroimidazole)dichloroplatinum(II) (NIPt)] were prepared in our laboratory as previously described (Teicher *et al.*, 1985).

### Cell line

The SCC-25 cell line was derived from the biopsy of a human squamous cell carcinoma of the tongue and was established and characterized initially by J.G. Rheinwald at this Institute (Rheinwald & Beckett, 1981). Monolayers were

Correspondence: B.A. Teicher.
Received 17th July 1986 and in revised form 20th November 1986.

maintained in Dulbecco's modified Eagle's medium (DMEM) supplemented with 5% foetal bovine serum, hydrocortisone (0.4 $\mu$g ml$^{-1}$) and antibiotics (Frei *et al.*, 1985; Teicher *et al.*, 1986). This cell line has a plating efficiency of 10–30% and a doubling time of 48 h (Frei *et al.*, 1985).

*Survival studies*

SCC-25 cells were grown to confluency, then the culture medium was renewed daily for 3 days and experiments were performed on the following day. After exposure to the drug or vehicle for 1 h, the cells were washed 2 times with 0.9% PBS and suspended by treatment with 0.125% trypsin/0.05% EDTA. The cells were plated in duplicate dishes at 3 dilutions for colony formation. After 2 weeks, the colonies were visualized by staining with crystal violet and colonies of 50 cells or greater were counted. The results are expressed as surviving fraction of treated cells compared to vehicle-treated control cells.

*Survival studies for PLDR*

Stationary phase cultures of SCC-25 cells were prepared as described above. The cells were exposed to 100 mU ml$^{-1}$ of bleomycin for 1 h at 37°C in fresh medium. The medium covering the monolayers before treatment (depleted medium) was retained and used to cover the cultures during the delay from subculture period. After treatment with bleomycin, the dishes were rinsed twice with PBS and depleted medium was added. Platinum complexes were added to the depleted medium for the duration of the delay to the time of subculture. The concentrations of platinum complexes were 0.5 $\mu$M CDDP, 10 $\mu$M Plant, 10 $\mu$M Plato, 10 $\mu$M and 25 $\mu$M NIPt and 10 $\mu$M and 25 $\mu$M Pt(terpy). Similar dishes which had not been treated with bleomycin were exposed to the platinum complexes for the same time periods to assess the cytotoxicity of the platinum complexes alone. Both treated and control dishes were held for 0, 1, 2, 4, 6 and 24 h at 37°C, then the cells were washed twice with PBS, suspended by trypsinization and counted by hemacytometer. Known numbers of cells were plated in duplicate dishes at 3 dilutions for colony formation as described above.

*Data analysis*

Quantitative analysis of survival curves was performed using the log-probit iterative least squares method of Litchfield and Wilcoxon (1949) as revised by Tallarida and Murray (1981). Calculations were performed on an Apple II+ microcomputer.

Recovery ratios ($R/R_0$) were calculated by dividing the surviving fraction immediately after drug exposure ($R_0$) into the surviving fraction of cells at each time post-treatment ($R$) corrected for the cytotoxicity of the inhibitor. A recovery ratio of 1.0 means no recovery or repair of damage and a ratio of greater than 1.0 means recovery from PLD (Barranco & Townsend, 1986).

### Results

The SCC-25 cell line is a well-established line derived from a human squamous carcinoma of the head and neck. The capacity of this cell line for PLDR following X-ray treatment has been documented with a 24 h recovery ratio ($R/R_0$) of 6.2 (Weichselbaum, 1984). Figure 1 shows the survival curve for stationary phase SCC-25 cells exposed to various concentrations of bleomycin. The survival curve is biphasic with an initial sensitive phase and a less sensitive second phase as is common for many cell lines (Twentyman, 1984). After stationary phase SCC-25 cells were exposed to 100 mU ml$^{-1}$ of bleomycin for 1 h, the drug was removed

**Figure 1** Survival curve for stationary phase SCC-25 cells treated with various concentrations of bleomycin for 1 h (●). *Inset*: PLD recovery (○) showing the loss of effectiveness of bleomycin (100 mU ml$^{-1}$) due to recovery from potentially lethal damage over 24 h. Error bars are s.e.

and the cells were allowed various periods for PLD recovery. After 24 h, the SCC-25 cells showed a recovery ratio ($R/R_0$) of 7.0 which corresponded to an immediate survival at a drug level of 27 mU ml$^{-1}$, a dose 3.7-fold less than the exposure concentration of 100 mU ml$^{-1}$. The recovery ratio for SCC-25 cells following bleomycin treatment increased rapidly at early time points. It was 2.5 at 1 h, 4.1 at 2 h and 5.3 at 4 h. The rate of recovery slowed after 4 h so that the recovery ratio was 6.1 at 6 h and 7.0 at 24 h.

Over the course of the first 4 h of PLD recovery, 0.5 $\mu$M CDDP was a very effective inhibitor of PLDR (Figure 2). For the first hour, while $R/R_0$ was 2.5 for bleomycin alone, with 0.5 $\mu$M CDDP the $R/R_0$ was 1.1. Between 2 and 4 h the $R/R_0$ was 1.6–1.8 with CDDP and 4.1–5.3 without CDDP. However, at the 6 h point the cell survival in the presence of CDDP increased, producing a recovery ratio of 3.9 with CDDP compared to 6.1 without CDDP. Plant (10 $\mu$M) and Plato (10 $\mu$M) produced PLD recovery inhibition patterns very similar to that of CDDP. After 1 h the recovery ratios in the presence of Plant and Plato were 1.1–1.3. Between 2 and 4 h, Plato gave recovery ratios of 1.8–2.3 and Plant gave recovery ratios of 1.6–1.9. By 6 h both Plato and Plant were less effective inhibitors than CDDP, producing recovery ratios of 5.3 and 4.4 respectively.

NIPt and Pt(terpy) were examined at both 10 $\mu$M and 25 $\mu$M for their ability to inhibit PLD recovery after bleomycin treatment (Figure 2). There was no significant difference between PLDR inhibitory effects by the different concentrations of these agents. After 1 h, NIPt gave a recovery ratio of 1.3–1.4, at 2–4 h the recovery ratio was 1.7–2.6 and at 6 h the recovery ratio was 3.6–3.8. Pt(terpy) gave recovery ratios of 1.3–1.6 after 1 h, 1.5–1.8 at 2–4 h and 3.5–4.0 at 6 h. With NIPt and Pt(terpy), there was a continuous increase in the recovery ratios compared to the more stepwise increase in recovery ratios with CDDP, Plant and Plato.

**Figure 2** *Panels a–c*: Survival of stationary phase SCC-25 cells treated with 100 μM bleomycin which were allowed various periods of time for PLD recovery (*). *Panel a*: Survival of these same cells exposed to 0.5 μM CDDP (●), 10 μM Plant (■) or 10 μM Plato (▲) during the PLDR recovery period. Survival of untreated cells exposed to 0.5 μM CDDP (○), 10 μM Plant (□) or 10 μM Plato (△) for the indicated time periods. *Panels b and c*: Survival of these same cells exposed to 10 μM NIPt (●), 25 μM NIPt (◆), 10 μM Pt(terpy) (▲) or 25 μM Pt(terpy) (■) during the PLDR recovery period. Survival of untreated cells exposed to 10 μM NIPt (○), 25 μM NUPt(terpy) (◇) or 25 μM Pt(terpy) (□) for the indicated time periods. Error bars are the s.e.

### Discussion

The significance of PLDR has remained controversial (Twentyman, 1984; Weichselbaum *et al.*, 1982; Weichselbaum *et al.*, 1984); however, it seems reasonable that drug combinations which inhibit the ability of tumour cells to repair significant portions of drug-induced damage will lead to improved clinical treatment. The ability of mammalian cells to recover from bleomycin-induced damage has been well-documented both *in vitro* and *in vivo* (Barranco & Townsend, 1986). The process can be inhibited with actinomycin D, ethanol and hyperthermia (Barranco, 1978; Twentyman, 1984) or under hypoxic conditions by misonidazole (Korbelik *et al.*, 1985). More recently it has been shown that some platinum complexes can inhibit the recovery of V79 cells from radiation-induced cell kill (O'Hara *et al.*, 1986). We have shown that CDDP and four other novel platinum complexes can inhibit to a moderate degree PLD recovery of stationary phase SCC-25 cells treated with bleomycin. Increased repair is one possible mechanism of resistance to chemotherapeutic agents (Teicher *et al.*, 1986). Drug combinations which can inhibit these repair processes and overcome resistance would be of great value in the clinic. The platinum complexes used in this study have been previously shown to potentiate the cytotoxicity of mitomycin C, especially in hypoxic cells (Teicher *et al.*, 1986). It is possible, therefore, to speculate on the possibility of a drug combination including euoxic and hypoxic selective drugs such as bleomycin/mitomycin C and at low doses a platinum complex which may inhibit potentially lethal damage recovery from bleomycin cytotoxicity and potentiate mitomycin C cytotoxicity. Our data suggest new avenues for therapeutic research in SCCHN. Since repair of SCC-25 cells after treatment with bleomycin begins 1–2 h after treatment, fractionated doses or long term infusion of bleomycin as proposed by Umezawa (1982) or intermittent large dose treatment may be superior. Also, since the addition of relatively non-toxic doses of the platinum complexes to bleomycin resulted in a significant reduction of PLD repair, the addition of other chemotherapeutic drugs such as CDDP may further enhance the therapeutic efficacy of bleomycin treatment of tumours.

This work was supported by NIH fellowship No. 5F32-CA-07821-02 (SAH), NCI grant No. RO1-CA36508-03 (BAT) and a grant from Johnson-Matthey Inc., West Chester, PA (BAT).

### References

BARRANCO, S.C. (1978). A review of the survival and cell kinetics effects of bleomycin. In *Bleomycin: Current Status and New Developments*, Carter *et al.* (eds) p. 151. Academic Press: New York.

BARRANCO, S.C. & TOWNSEND, C.M. JR. (1986). Loss in cell killing effectiveness of anticancer drugs in human gastric cancer clones due to recovery from potentially lethal damage *in vitro*. *Cancer Res.*, **46**, 623.

BOEHEIM, K. & SPOENDLIN, H. (1986). 3-year results of combined modality therapy in locally advanced, resectable squamous cell carcinoma of the head and neck. *Acta Otolaryng.* (*Stockh*), (in press).

ERVIN, T.J., WEICHSELBAUM, R., MILLER, D., MESHAD, M., POSNER, M. & FABIAN, R. (1981). Treatment of advanced squamous cell carcinoma of the head and neck with cisplatin, bleomycin and methotrexate (PBM). *Cancer Treat. Rep.*, **65**, 787.

FREI, E. III, CUCCHI, C.A., ROSOWSKY, A. & 5 others (1985). Alkylating agent resistance *in vitro* studies with human cell lines. *Proc. Natl Acad. Sci. USA*, **82**, 2158.

GLICK, J.H., MARCIAL, V., RICHTER, M., VELEZ GARCIA, E. (1980). The adjuvant treatment of inoperable stage III and IV epidermoid carcinoma of the head and neck with platinum and bleomycin infusions prior to definitive radiotherapy: An RTOG pilot study. *Cancer*, **46**, 1919.

HAHN, G.M. & LITTLE, J.B. (1972). Plateau phase culture of mammalian cells: An *in vitro* model for human cancer. *Curr. Top. Rad. Res. Quart.*, **8**, 39.

HONG, W.K., BHUTANI, R., SHAPSHEY, S.M. & STRONG, S. (1980). Induction chemotherapy of advanced previously untreated squamous cell head and neck cancer with *cis*platin and bleomycin. In *Cisplatin: Current Status and Developments*, In Prestayko *et al.* (eds) p. 431. Academic Press: New York.

KORBELIK, M., PALCIC, B. & SKARSGARD, L.D. (1985). Bleomycin and misonidazole cytotixicity. *Br. J. Cancer*, **51**, 499.

LITCHFIELD, J.T. & WILCOXON, F. (1949). A simplified method of evaluating dose-effect experiments. *J. Pharmacol. Exp. Therap.*, **96**, 99.

O'HARA, J.A., DOUPLE, E.B. & RICHMOND, R.C. (1986). Enhancement of radiation-induced cell kill by platinum complexes (carboplatin and iproplatin) in V79 cells. *Int. J. Radiat. Oncol. Biol. Phys.*, **12**, (in press).

RAY, G.R., HAHN, G.M., BAGSHAW, M.A. & KURKJIAN, S. (1973). Cell survival and repair of plateau phase cultures after chemotherapy: Relevance to tumor therapy and to the *in vitro* screening of new agents. *Cancer Chemother. Rep.*, **57**, 473.

RHEINWALD, J.G. & BECKETT, M.A. (1981). Tumorigenic keratinocyte lines requiring anchorage and fibroblast support cultured from human squamous cell carcinomas. *Cancer Res.*, **41**, 1657.

SHAH, P.M., SHUKLA, S.N., PATEL, K.M., BABOO, H.A. & PATEL, D.D. (1981). Effect of bleomycin-radiotherapy combination in management of head and neck squamous cell carcinoma. *Cancer*, **48**, 1106.

SPAULDING, M.B., KAHN, A., DE LOS SANTOS, R., KLOTCH, D. & LORE, J.M. (1982). Adjuvant chemotherapy in head and neck cancer: An update. *Am. J. Surg.*, **144**, 432.

TALLARIDA, R.J. & MURRAY, R.B. (1981). *Manual of Pharmacologic Calculations with Computer Programs.* Springer-Verlag, New York.

TEICHER, B.A., CUCCHI, C.A., LEE, J.B., FLATOW, J.L., ROSOWSKY, A. & FREI, E. III. (1986). Alkylating agents: *In vitro* studies of cross resistance patterns. *Cancer Res.*, **46**, 4379.

TEICHER, B.A., ROCKWELL, S. & LEE, J.B. (1985). Radiosensitization of EMT6 cells by four platinum complexes. *Int. J. Radiat. Oncol. Biol. Phys.*, **11**, 937.

TURRISI, A.T. III, ROZENCWIEG, M., VON HOFF, D.D. & MUGGIA, F.M. (1978). The role of bleomycin in the treatment of advanced head and neck cancer. In *Bleomycin: Current Status and New Developments*, Carter *et al.* (eds) p. 151. Academic Press: New York.

TWENTYMAN, P.R. (1984). Bleomycin: Mode of action with particular reference to the cell cycle. *Pharmacol. Therap.*, **23**, 417.

UMEZAWA, M. (1982). Principles of antitumor antibiotic therapy. In *Cancer Medicine*, Holland & Frei (eds) p. 860. Lea Febiger: Philadelphia.

WEICHSELBAUM, R.R. (1984). The role of DNA repair processes in the response of human tumors to fractionated radiotherapy. *Int. J. Radiat. Oncol. Biol. Phys.*, **10**, 1127.

WEICHSELBAUM, R.R., DAHLBERG, W., LITTLE, J.B. & 4 others (1984). Cellular X-ray repair parameters of early passage squamous cell carcinoma lines derived from patients with known responses to radiotherapy. *Br. J. Cancer*, **49**, 595.

WEICHSELBAUM, R.R., SCHMIT, A. & LITTLE, J.B. (1982). Cellular repair factors influencing radiocurability of human malignant tumors. *Br. J. Cancer*, **45**, 10.

WITTES, R., HELLER, K., RANDOLPH, V. & 8 others (1979). *Cis*-diamminedichloroplatinum(II)-based chemotherapy as initial treatment of advanced head and neck cancer. *Cancer Treat. Rep.*, **63**, 1533.